# Segregation of Multimodal Inputs Into Discrete Midbrain Compartments During an Early Critical Period

**DOI:** 10.3389/fncir.2022.882485

**Published:** 2022-04-07

**Authors:** Jacob M. Weakley, Erin K. Kavusak, Julianne B. Carroll, Mark L. Gabriele

**Affiliations:** Department of Biology, James Madison University, Harrisonburg, VA, United States

**Keywords:** development, inferior colliculus, multisensory, GAD, anterograde, modules, matrix

## Abstract

The lateral cortex of the inferior colliculus (LCIC) is a multimodal subdivision of the midbrain inferior colliculus (IC) that plays a key role in sensory integration. The LCIC is compartmentally-organized, exhibiting a series of discontinuous patches or modules surrounded by an extramodular matrix. In adult mice, somatosensory afferents target LCIC modular zones, while auditory afferents terminate throughout the encompassing matrix. Recently, we defined an early LCIC critical period (birth: postnatal day 0 to P12) based upon the concurrent emergence of its neurochemical compartments (modules: glutamic acid decarboxylase, GAD+; matrix: calretinin, CR+), matching Eph-ephrin guidance patterns, and specificity of auditory inputs for its matrix. Currently lacking are analogous experiments that address somatosensory afferent shaping and the construction of discrete LCIC multisensory maps. Combining living slice tract-tracing and immunocytochemical approaches in a developmental series of GAD67-GFP knock-in mice, the present study characterizes: (1) the targeting of somatosensory terminals for emerging LCIC modular fields; and (2) the relative separation of somatosensory and auditory inputs over the course of its established critical period. Results indicate a similar time course and progression of LCIC projection shaping for both somatosensory (corticocollicular) and auditory (intracollicular) inputs. While somewhat sparse and intermingling at birth, modality-specific projection patterns soon emerge (P4–P8), coincident with peak guidance expression and the appearance of LCIC compartments. By P12, an adult-like arrangement is in place, with fully segregated multimodal afferent arrays. Quantitative measures confirm increasingly distinct input maps, exhibiting less projection overlap with age. Potential mechanisms whereby multisensory LCIC afferent systems recognize and interface with its emerging modular-matrix framework are discussed.

## Introduction

An essential role of the nervous system is to encode and integrate various sensory stimuli. Such processing requires highly-ordered network configurations that coordinate converging inputs from distinct systems. The inferior colliculus (IC) is a strategically positioned midbrain hub that receives an array of top-down and bottom-up afferents of multisensory origin. Within its lateral cortex (LCIC) single units respond to a variety of stimuli (Aitkin et al., 1978, 1981; Gruters and Groh, 2012), suggesting an underlying neuroanatomical substrate capable of multisensory processing. In adult mice, input channels target discrete regions of the LCIC in a modality-specific manner (Lesicko et al., 2016). Somatosensory projections terminate as a series of discontinuous patches or modules that span LCIC layer 2. In contrast, auditory inputs exhibit complementary patterns, preferentially targeting the surrounding LCIC matrix (layers 1, 3, and intermodular zones). A recent study examining intrinsic LCIC circuits shows that local connectivity is largely confined to its own compartment (module or matrix), although unidirectional flow from matrix regions that receive auditory inputs into somatosensory-rich modular zones also occurs (Lesicko et al., 2020). Such intercompartmental connectivity likely underlies its reported multisensory capabilities that in turn inform precise response behaviors.

To date little is known about the development of multimodal LCIC afferent systems. Previous work from our lab defined an early postnatal LCIC critical period (birth: postnatal day 0, P0 through P12) based on the emergence of its characteristic compartmental framework (Chernock et al., 2004). A host of neurochemical stains reveal that while not apparent at birth, a discrete microarchitecture quickly emerges (Dillingham et al., 2017). Most noteworthy from the identified markers are GAD (glutamic acid decarboxylase) which reliably highlights LCIC modules, and calretinin (CR) which labels the extramodular matrix. Temporally correlated with the emergence of LCIC neurochemical compartments is the transient expression of matching Eph-ephrin guidance patterns (Gay et al., 2018; Stinson et al., 2021). Expression of both EphA4 and ephrin-B2 align with its developing modularity, while ephrin-B3 expression is complementary and restricted to the surrounding matrix.

We hypothesized that an early period of projection shaping exists whereby somatosensory and auditory afferents target appropriate LCIC compartments. Recently, we characterized the development of auditory inputs to the LCIC arising from its neighboring central nucleus (CNIC, Lamb-Echegaray et al., 2019). While initially diffuse at birth, an early projection specificity for the LCIC matrix soon develops. This mapping preference of LCIC auditory afferents appears adult-like by P12, thus sharing a similar critical period defined for its emerging micro-organization and matching Eph-ephrin guidance patterns. Here, we combine anterograde tract-tracing approaches in living preparations of developmental GAD67-GFP tissue with immunocytochemical methods to: (1) determine whether somatosensory inputs follow a similar developmental progression in targeting LCIC modular zones; and (2) visualize and quantify the segregation of two multimodal LCIC afferent patterns (somatosensory: corticocollicular; auditory: from the CNIC) with respect to each other. Results indicate a model in which initially overlapping projection distributions refine and segregate into modality-specific compartments during an early postnatal window. Potential mechanisms that may instruct discretely-organized LCIC multisensory maps are discussed.

## Materials and Methods

### Animals

Experiments were performed on neonatal GAD67-GFP mice (P0, P4, P8, and P12; *n* = 36, consisting of at least three mice at each age for both single- and dual tracer studies). The GAD67-GFP knock-in line has previously been validated in our lab (Gay et al., 2018) and allows for easy visualization of GAD-positive LCIC modules (Lesicko et al., 2016, 2020; Gay et al., 2018; Lamb-Echegaray et al., 2019; Stinson et al., 2021; Brett et al., 2022). Specifics concerning the generation of the GAD67-GFP (Δneo) line are described elsewhere (Tamamaki et al., 2003; permission granted by Dr. Yuchio Yanagawa, Gunma University Graduate School of Medicine, Gunma, Japan). Heterozygous GAD-GFP males were crossed with C57BL/6J females and GFP-expressing progeny were identified before P4 using a Dark Reader Spot Lamp Goggle visualization system (Clare Chemical Research^1^, Dolores, CO, Cat# SL10S). Equal numbers of males and females were used in the described experimentation and no sex-specific differences were noted. All procedures were performed in keeping with the US National Research Council’s *Guide for the Care and Use of Laboratory Animals* and received prior approval by the Institutional Animal Care and Use Committee (Protocol No. 20-1421).

### Anterograde Tracing in Living Preparations

Following an overdose of ketamine (200 mg/kg) and xylazine (20 mg/kg), mice at designated stages were perfused with chilled, oxygenated (95% O_2_, 5% CO_2_) artificial cerebrospinal fluid (aCSF; in mM; 126 NaCl, 3 KCl, 1.25 NaH_2_PO_4_, 10 dextrose, 20 NaHCO_3_, 1.2 MgSO_4_, 2.5 CaCl_2_, pH 7.4). Brains were blocked in the coronal plane (just rostral to somatosensory cortex and just caudal to the midbrain) and varied in total thickness depending upon age. Under a dissecting microscope, gross placements of biocytin crystal (B1758, Sigma-Aldrich, St. Louis, MO) were made in somatosensory cortex at varying depths depending on age to maximize labeling of layer 5/6 corticocollicular projections. In double-labeling studies, the cerebellum was then removed to facilitate deposition of a 10,000 MW dextran (D22904, AlexaFluor 647 direct conjugate, ThermoFisherScientic, Waltham, MA) in the CNIC ipsilateral to the cortical biocytin placement. Tissue blocks were bubbled at room temperature in aCSF for 18–24 h to allow for complete filling of terminal endings in the LCIC. Prior to sectioning, tissue was postfixed and cryoprotected in a series of 4% paraformaldehyde solutions with increasing concentrations (10%–30%) of sucrose.

### Tissue Processing and Immunocytochemistry

A rostrocaudal series of sections were cut at 50 μm on a sliding freezing microtome and collected in 0.1 M phosphate buffered saline (PBS, pH 7.4). Free-floating sections were rinsed three times for 5 min in PBS. In experiments combining tract-tracing with a matrix marker, a 2.5 h DyLight 549 streptavidin step (1:200, SA-5549, Vector Laboratories, Burlingame, CA, RRID:AB_2336408) for visualizing biocytin always preceded subsequent CR immunostaining. Following another round of PBS rinses, sections were blocked in 5% normal donkey serum (NDS) in PBS for 30 min. Tissue was then incubated in anti-CR primary made in rabbit (1:250, CR 7697, Swant, Burgdorf, Switzerland, RRID:AB_2619710) for 40 min at room temperature and then overnight at 4°C. An Alexa Fluor 350 donkey anti-rabbit IgG (1:25, A10039, Thermo Fisher Scientific, Waltham, MA, RRID:AB_2534015) was then applied prior to a final series of PBS rinses. Sections were mounted on charged slides, coverslipped with Pro Long Diamond (P36970, Thermo Fischer Scientific, Waltham, MA), and stored in the dark until imaging.

### Image Acquisition and Quantitative Methods

Aside from documentation of somatosensory cortex tracer placements, data collection focused exclusively on the ipsilateral IC, as corticofugal projections to the contralateral LCIC are sparse. Verification of accurate cortical and CNIC tracer deposits were made using age-matched sections in The Atlas of the Developing Mouse Brain (Paxinos et al., 2007). Sections throughout the rostrocaudal extent of the IC were imaged using epiflourescent microscopy (Nikon Eclipse Ti-2 microscope equipped with a monochrome, Hamamatsu ORCA-Flash 4.0 V3 sCMOS camera, Japan; PlanApo objectives 10× : NA = 0.30, 20× : NA = 0.75, and 40× : NA = 1.30). Filter sets (Chroma Technology, Bellows Falls, VT) were designed with careful attention to the spectra of the various fluorophores to ensure no cross-channel bleed through (Alexa Fluor 350 filter set: excitation 381–403 nm emission 417–477; GFP filter set: excitation 446–486 emission 500–550; biocytin-streptavidin DyLight 549 filter set: excitation 542–566 emission 582–636; dextran AlexaFluor 647 filter set: excitation 593–643 emission 663–733. An extended depth of field (EDF) algorithm was used to generate two-dimensional images from acquired *Z*-stacks (Elements Software; Nikon) using only focused portions of each optical slice. Monochrome channel acquisitions were pseudocolored accordingly (blue: CR, green: GAD, biocytin: red, dextran: cyan) and saved as lossless JPEG2000 files.

Quantification of somatosensory afferent pattern alignment with emerging LCIC compartments, as well as assessments of multisensory pattern segregation, focused on mid-rostrocaudal regions of the LCIC where the modular-matrix framework was most readily apparent. Data from a minimum of three mice were analyzed at each age and multiple sections (a minimum of two) were sampled to account for any potential regional LCIC variability. LCIC layer 2 sampling and generation of brightness plot profiles has been previously described in detail (Wallace et al., 2016; Dillingham et al., 2017; Gay et al., 2018; Lamb-Echegaray et al., 2019; Stinson et al., 2021; Brett et al., 2022). In brief, separate image channels were converted to grayscale and exported as uncompressed TIFFS prior to importing into ImageJ^2^ (NIH, Bethesda, MD, RRID:SCR_003070). A freehand tool (line thickness 100 μm) was used to sample LCIC layer 2 from ventral-to-dorsal bisecting GAD-positive modules. Sampling contours were approximated through presumptive LCIC layer 2 in P0 mice, as GAD-defined modules are not easily discerned at this earliest postnatal time point. An ROI function was employed to apply the same sampling contour to each subsequent channel sampled of the same image. Generated signals (single tracer studies: somatosensory vs. GAD/CR, dual tracer studies: somatosensory vs. auditory) were compiled as multi-channel brightness profiles to visualize relative signal overlap/non-overlap. Raw data values for each waveform were exported for cross-correlation and kurtosis analyses described below. The latter analysis used F_S_ and F_A_ to refer to sampled somatosensory and auditory fluorescence values, respectively.

Cross-correlation analyses (Microsoft Excel, Redmond, WA) were performed to numerically quantify the similarity of two signal patterns relative to each other (e.g., somatosensory and GAD, or somatosensory and CR). Cross-correlation function y-intercepts (i.e., no spatial shift or relative displacement) serve as an objective measure for assessing signal matching/mismatch. Values range from +1.0 to −1.0, with higher values indicating strong signal overlap, whereas lower values indicate waveforms that are non-overlapping or out-of-phase. Single factor ANOVA assessed similarity among developmental stages. Based on ANOVA high confidence dissimilarity (*p* < 0.05), subsequent comparisons between age pairs were made using independent, two-tailed Student’s *t*-tests assuming unequal variance (Type 3) with statistical significance (*p* < 0.05). Similar *t*-tests (albeit one-tailed) were performed comparing y-intercepts of somatosensory vs. GAD and somatosensory vs. CR signal pairings.

To quantify the extent of segregation of developing multimodal LCIC inputs, we build upon established unbiased methods applied to emerging retinogeniculate patterns (Torborg and Feller, 2004; Jaubert-Miazza et al., 2005; Torborg et al., 2005). These previous methods processed afferent data in four main stages: (1) a “rolling ball” algorithm correcting for uneven background; (2) logarithmic ratios (*R* values) of ipsilateral and contralateral fluorescence intensities, F_I_ and F_C_ respectively; *R* = log_10_(F_I_/F_C_); (3) *R* value histograms revealing the extent of segregation in their shape; and finally (4) variances of *R*-distributions quantifying the extent of segregation in a single metric. Typically, overlapping patterns produce unimodal *R* distributions, while decreased projection overlap (or segregation) produce bimodal *R* distributions. Testing for overlap/segregation therefore requires quantifying the shape of *R* distributions.

Accordingly, we also perform the same first three stages, but in the last stage employ a different *R* distribution statistic (kurtosis rather than variance) to quantify *R* distribution shape to assess relative input separation. Variance is the second central moment (i.e., average squared deviations from the mean) and a measure of distribution spread. Kurtosis is the fourth central moment (i.e., average fourth power deviations from the mean; normalized by variance) and a direct measure of distribution shape. Unimodal distributions yield more positive kurtosis values, while bimodal distributions yield more negative kurtosis values. In sum, we also process our dual tract-tracing data in four stages: (1) applying a “rolling ball” filter in ImageJ (diameter = 20 pixels) correcting for uneven background fluorescence (i.e., regions of the LCIC exhibiting no axonal labeling); (2) logarithmic ratios (*R* values) combining fluorescence intensities of somatosensory and auditory inputs, F_S_ and F_A_ respectively; *R* = log_10_(F_S_/F_A_); (3) histograms of *R* values showing extent of segregation in their shape; and (4) kurtoses of *R*-distributions to quantify extent of segregation in single statistics.

## Results

### Development and Quantification of Descending Somatosensory Projections Targeting LCIC Modules

**Figure 1:**
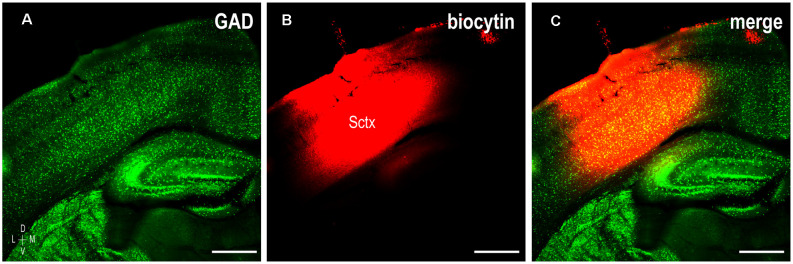
Representative tracer placement labeling somatosensory corticocollicular projections in a P12 mouse. GAD labeling (**A**, green) and biocytin crystal placement (**B**, red) encompassing deep output layers of somatosensory cortex (Sctx, **C**, merge). Scale bars = 500 μm.

**Figure 2:**
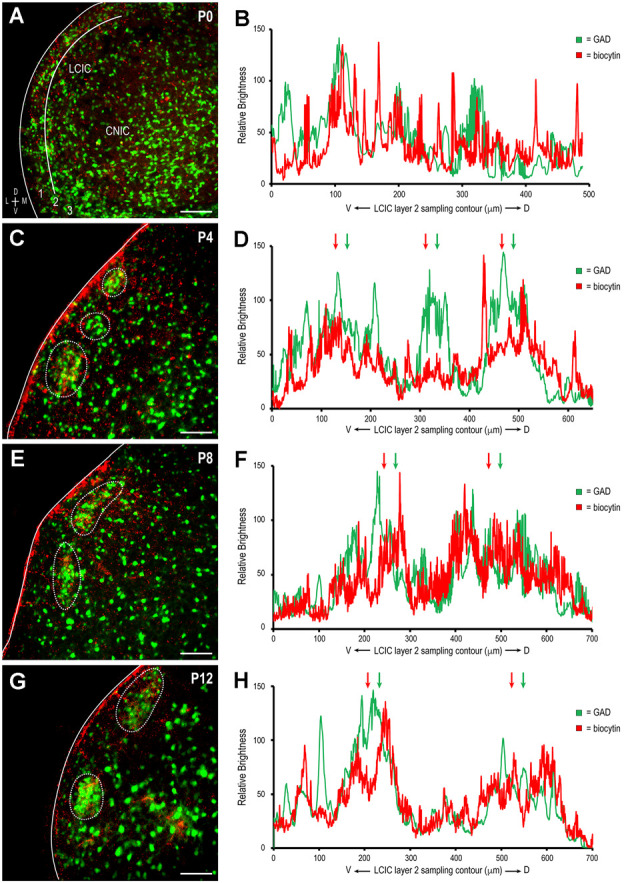
Development of descending somatosensory modular projection patterns in the LCIC. Biocytin-labeled terminals (red) with emerging GAD modules (green) at P0 **(A,B)**, P4 **(C,D)**, P8 **(E,F)**, and P12 **(G,H)**. Note the increased specificity and alignment with age of patchy terminal fields within modular confines (dashed contours in **C,E,G**) further evidenced by increased signal overlap in corresponding brightness profiles (arrows in **D,F,H**). LCIC layers = 1, 2, and 3. Scale bars = 100 μm.

To determine whether somatosensory inputs to the LCIC share the same critical period defined for auditory inputs (Lamb-Echegaray et al., 2019) and its emerging compartments, unilateral biocytin crystal placements were made in somatosensory cortex (Figure 1) in a developmental series of GAD67-GFP living preparations. Given the sparse nature of the contralateral corticocollicular pathway, analyses focused exclusively on the LCIC ipsilateral to the tracer placement. Terminals arising from somatosensory cortex were present in the LCIC at birth and diffusely distributed (Figure 2A), lacking any regional specificity. GAD-positive modules at this age are not readily apparent (Figure 2B), in keeping with previous reports (Dillingham et al., 2017; Gay et al., 2018; Lamb-Echegaray et al., 2019; Stinson et al., 2021; Brett et al., 2022). As layer 2 modular fields emerge at P4 and continue to develop through P12, a preference of somatosensory endings for these defined zones becomes increasingly apparent (Figures 2C–H). By P12, projection specificity appears adult-like, with elaborate arborizations within modular confines and little evidence of significant labeling in the surrounding matrix.

**Figure 3:**
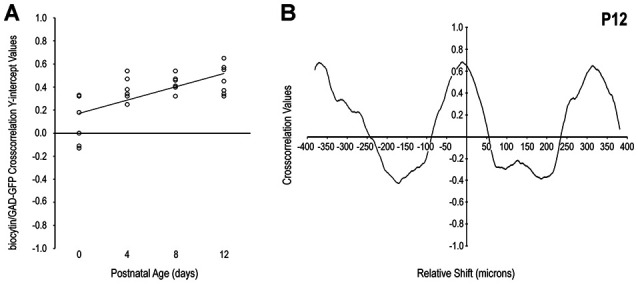
Quantification of LCIC somatosensory patterns relative to GAD modular expression over its early critical period. The positively sloped linear regression of cross-correlation y-intercept values with age **(A)** supports the observed developmental trend that somatosensory afferents become increasingly modular and align with emerging GAD-positive layer 2 modules. A representative P12 cross-correlation function **(B)** exhibits a distinctly positive y-intercept (significant signal matching) with nearly symmetrical off-origin maxima and minima that indicate generally similar periodic patterns at this age.

Qualitative observations of increased matching of somatosensory and GAD patterns with age (i.e., in-phase signal waveform oscillations, arrows in Figures 2D,F,H) were confirmed with cross-correlation analyses. The positively sloped linear regression of y-intercept values plotted by developmental stage supports the notion that initially dispersed somatosensory inputs become increasingly specific for LCIC modules (Figure 3A). Single factor ANOVA showed significant mean dissimilarity among age groups (*p* < 0.001). Thus, *t*-tests comparing age pairs were performed that showed cross-correlation y-intercept values statistically different for P0 vs. P4 (*p* = 0.019), P0 vs. P8 (*p* = 0.009), and P0 vs. P12 (*p* = 0.005), but not other age pairs (*p* > 0.05). Off-origin cross-correlation function maxima and minima occur at certain relative spatial shifts between the two series, signifying aligned and mis-aligned signals, respectively. A representative P12 cross-correlation function (Figure 3B) shows a distinctly positive y-intercept (i.e., strong signal matching with no spatial shift) and pronounced off-origin peaks and troughs that reflect the periodic nature of the LCIC modular-matrix framework at this age. Taken together, these data suggest a similar period of projection shaping for top-down somatosensory inputs that gradually sharpen and overlap emerging LCIC modular domains.

**Figure 4:**
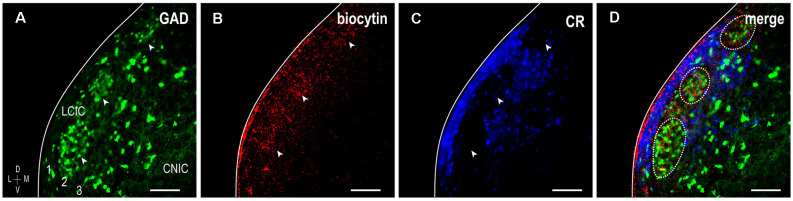
Somatosensory corticocollicular projections specifically target GAD modules by P12, and are complementary to the CR-positive matrix. GAD-positive modules are easily discerned at this age (**A**, arrowheads) and align with patchy somatosensory terminal fields (**B**, arrowheads), as well as layer 2 modular voids evident in CR staining of the matrix (**C**, arrowheads). A digital merge **(D)** of the separated channels provides definitive evidence of highly refined projection patterns reminiscent of those previously described in adult mouse (see Lesicko et al., 2016). Scale bars = 100 μm.

**Figure 5:**
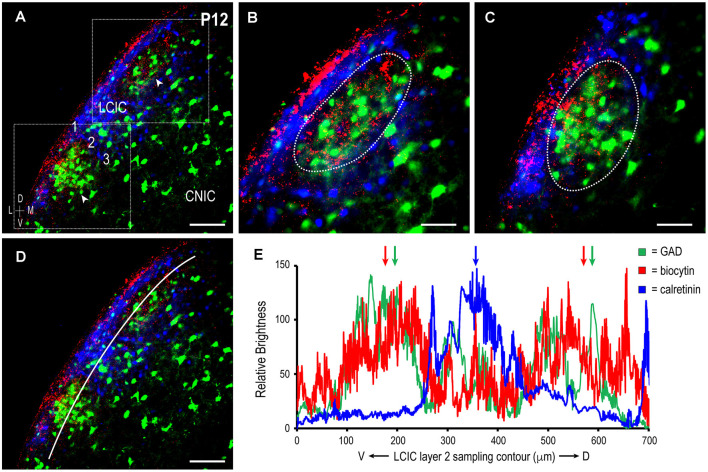
Descending projections arising from somatosensory cortex target discrete LCIC modular fields and avoid the matrix in another P12 mouse. Digital merge of GAD (green), biocytin (red), and calretinin (blue) in a mid-rostrocaudal LCIC section **(A)**. Higher magnification images **(B,C)** of corresponding insets boxes in **(A)** show highly refined terminal patterns in two adjacent LCIC modules. Note that axonal labeling is most concentrated within GAD-defined modules (dashed contours), and sparse at this time point in the encompassing CR-positive matrix. ROI layer 2 LCIC sampling for each channel **(D)** yields signal periodicities in **(E)** that exhibit either strong overlap (somatosensory and GAD, green and red arrows) or striking mismatch (CR, blue arrow, relative to somatosensory and GAD). Scale bars in **(A,D)** = 100 μm, **(B,C)** = 50 μm.

To further confirm the establishment of highly refined somatosensory inputs for LCIC modules by its critical period closure (P12), analogous labeling studies were performed coupled with immunostaining for the established matrix marker, calretinin. As anticipated, concentrated somatosensory terminal fields overlapping layer 2 GAD cell clusters filled voids in the encompassing CR-positive matrix (Figure 4, arrowheads). This complementary patterning was reliably observed in additional P12 mice (Figure 5A). Higher magnification reveals dense somatosensory terminal fields sculpted such that they nearly exclusively reside within modular confines (Figures 5B,C, dashed contours). Three-channel LCIC layer 2 sampling and corresponding brightness plot profiles illustrate oscillatory waveforms, with matching somatosensory and GAD signals that are mismatched with CR fluctuations (Figures 5D,E, arrows).

**Figure 6:**
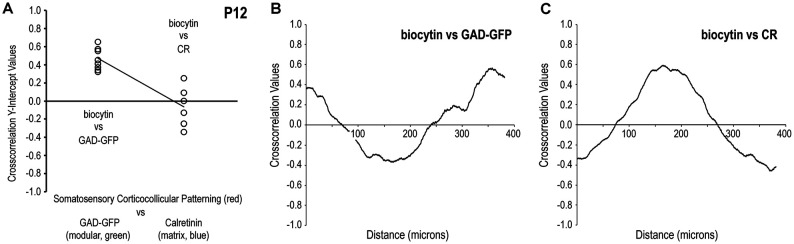
Quantification of discrete somatosensory projection patterns with respect to the LCIC modular-matrix framework at P12. Significantly different cross-correlation y-intercept values for biocytin and GAD vs. biocytin and CR (*p* < 0.001) confirm projection patterns that overlap modular zones and are complementary to the LCIC matrix **(A)**. Linear regression shows clear disparity of y-intercept values for the two conditions. A representative P12 cross-correlation function of somatosensory and GAD labeling in **(B)** contrasts with that of somatosensory and CR labeling in **(C)**. Namely, the function shapes are essentially the inverses of each other.

Comparisons of cross-correlation y-intercept values for somatosensory/GAD vs. somatosensory/CR signal combinations further demonstrate discrete afferent patterns at P12 that target LCIC modules (Figure 6). Tightly clustered positive y-intercepts for somatosensory/GAD labeling indicate reliable signal matching or registry. In contrast, more negative cross-correlation y-intercept values for the same axonal distributions with respect to CR matrix labeling confirm non-overlapping patterns or signal mismatch (Figure 6A). Such quantitative findings support our qualitative observations (Figures 4, 5) that somatosensory terminals spatially align with modular domains and are offset from the encompassing matrix. The y-intercept median and standard deviation for biocytin and GAD signals were + 0.45 and 0.12, respectively, whereas that for biocytin and CR signals were—0.07 and 0.22. Not surprisingly given these dissimilar medians and overall distributions, these two data sets proved to be statistically different from each other (*p* < 0.001). A representative P12 cross-correlation function for biocytin and GAD shows a positive y-intercept (i.e., strong overlap at zero shift) with an off-origin trough and peak indicative of strong signal mismatching and matching respectively at increasing relative shifts (Figure 6B). A contrasting P12 cross-correlation function for biocytin and CR exhibits a negative y-intercept (i.e., non-overlap at zero shift) with an off-origin peak and trough indicative of subsequent strong signal matching and mismatching with increasing relative shifts (Figure 6C).

### Early Segregation of Multisensory Input Maps Into Discrete LCIC Compartments

**Figure 7:**
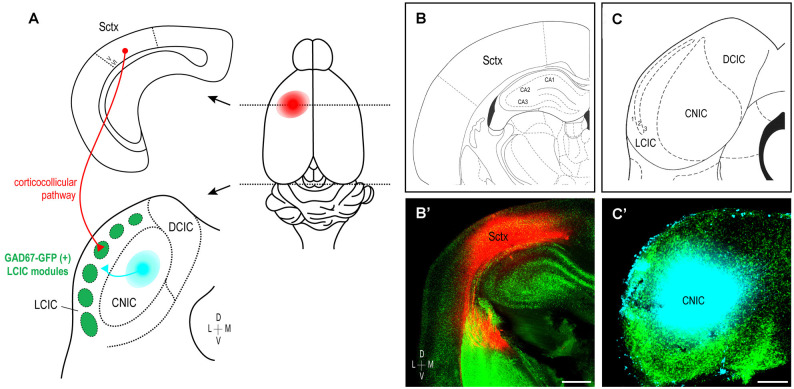
Experimental design schematic and verification of tracer placements. Simultaneous labeling of somatosensory and auditory LCIC afferents in early postnatal GAD67-GFP mice **(A)**. Biocytin placements made in the left somatosensory cortex label descending corticocollicular projections arising from layers 5/6 (red). Dextran positioned in the left CNIC labels auditory inputs (cyan) to the neighboring LCIC. Photomicrographs of coronal sections in a P4 mouse matched with corresponding atlas plates confirm accurate dye placements **(B,B’,C,C’)**. Scale bar in **(B’)** = 500 μm, in **(C’)** = 200 μm.

These findings, taken together with previous work from our lab characterizing developing auditory patterns (Lamb-Echegaray et al., 2019), suggest multimodal LCIC inputs share a similar critical period of shaping that yields complementary projection arrangements. Furthermore, they point towards emergence of discrete maps from initially intermingling distributions that undergo a process of segregation. To directly test such notions, we performed dual tracer studies simultaneously labeling somatosensory (corticocollicular) and auditory (arising from the CNIC) inputs in GAD67-GFP mice (Figure 7A) over the defined critical period (P0–P12). In each case, coronal sections containing placement centers for both dyes were documented and matched with corresponding atlas plates to verify tracer location (Figures 7B,B’,C,C’; plates modified for simplicity from Paxinos et al., 2007). As in the single-labeling experiments, placements centered in somatosensory cortex were made sufficiently deep to include corticocollicular cells of origin located in layers 5/6 (Figures 7B,B’, see Slater et al., 2013; Stebbings et al., 2014). CNIC placements were appropriately large to fill this subdivision, while not bleeding into the neighboring LCIC or DCIC (Figures 7C,C’).

**Figure 8:**
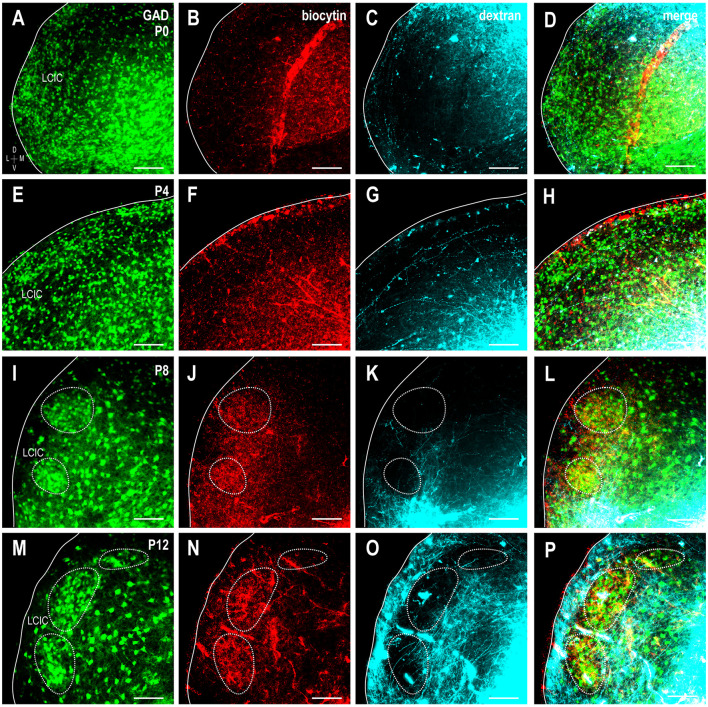
Shaping of somatosensory corticocollicular (red) and auditory CNIC (cyan) inputs with respect to emerging LCIC modularity (green). Developmental progression shown for stages P0 **(A–D)**, P4 **(E–H)**, P8 **(I–L)**, and P12 **(M–P)**. Projections are unorganized at birth and often remain so at P4 as LCIC compartments are just emerging. By P8, GFP-positive modules are evident (dashed contours) as somatosensory and auditory terminals appear to separate. Adult-like, fully segregated multisensory afferent patterns are reliably observed at P12. Scale bars = 100 μm.

**Figure 9:**
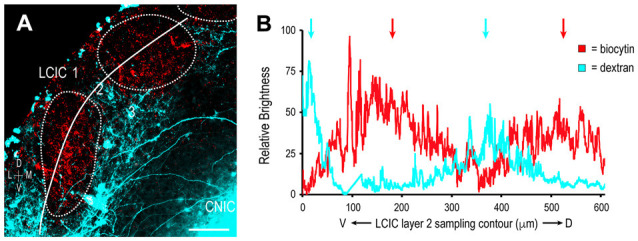
Segregation of multimodal LCIC projection patterns at P12. Somatosensory inputs (red) target layer 2 modules (dashed contours), while auditory inputs (cyan) distribute throughout the encompassing matrix **(A)**. A corresponding brightness plot profile confirms non-overlapping terminal patterns (arrows) at P12 **(B)**. Scale bar = 100 μm.

At birth, both somatosensory and auditory terminals are present in the LCIC, albeit sparse and unorganized (Figures 8A–D). Results at P4 were somewhat variable, with some cases still exhibiting intermingling patterns and inconclusive modules (Figures 8E–H), while others show clear indications of an emerging modularity and initial projection separation. These findings suggest P4 as a pivotal timepoint for the developing LCIC compartmental framework and its interfacing multimodal afferents. By P8, GAD-positive modules are easily discerned and projection segregation is in progress, as somatosensory inputs preferentially terminate within modular zones and auditory inputs the surrounding matrix (Figures 8I–L). By P12, the process of segregation is largely complete, with adult-like projection patterns that terminate almost exclusively within their respective compartments (Figures 8M–P, Supplementary Figure 1). Sampling of tracer channels at P12 consistently generate periodic waveforms with out-of-phase signal fluctuations, signifying fully segregated multimodal patterns (Figure 9).

### Quantification Reveals Progressive Input Segregation Over Critical Period

**Figure 10:**
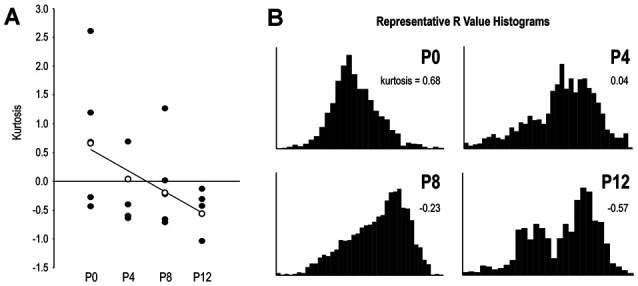
*R* distribution shape by its kurtosis statistic reveals progressive projection separation during the defined LCIC critical period. Linear regression of kurtosis values by age highlights the developmental trend of increasingly segregated multimodal inputs **(A)**. Open data markers in **(A)** closest to the regression line at each age were chosen for plotting representative *R* value histograms in **(B)**. Note distinct unimodal character of the P0 distribution as contrasted with the distinct bimodal distribution at P12. P4 and P8 distributions show an emerging and growing tail leading to clear bimodality at P12.

The kurtosis statistic was used to quantify the shape of *R* distributions which were calculated from somatosensory/auditory fluorescence ratios. Generally higher kurtosis values at earlier ages, contrasting with negative kurtosis values at later ages, indicate gradual changes in distribution shape from unimodal to bimodal with increasing age (Figure 10). Since overlapping projections produce unimodal *R* distributions and segregated projections produce bimodal *R* distributions, this kurtosis trend demonstrates increasing input separation with age. Linear regression depicts this developmental trend (Figure 10A). Representative *R* value histograms at each age reveal the transition from unimodal (overlapping) to bimodal (non-overlapping) from P0 to P12 (Figure 10B).

## Discussion

The current study provides evidence that mapping of somatosensory inputs to discrete LCIC compartments occurs alongside that previously described for auditory inputs (Lamb-Echegaray et al., 2019). During this early critical period a process of segregation takes place such that inputs of different modalities recognize and target complementary zones of its emerging patch-matrix-like organization. At birth, prior to the earliest indications of any developing modularity, somatosensory and auditory inputs intermingle throughout the LCIC. From P4 to P8, coincident with the appearance of its discrete neurochemical compartments (Dillingham et al., 2017) and matching Eph-ephrin guidance patterns (Gabriele et al., 2011; Cramer and Gabriele, 2014; Wallace et al., 2016; Gay et al., 2018; Stinson et al., 2021), inputs of the two modalities begin to separate. By P12, inputs appear adult-like and fully segregated, with highly specific terminal fields that recognize LCIC compartmental boundaries and exhibit little evidence of any remaining projection overlap. Quantification of LCIC afferent patterns confirm this developmental trend that initially overlapping projection distributions soon yield non-overlapping, modality-specific input maps (i.e., somatosensory: modules, auditory: matrix).

Beyond the two inputs examined in the present study, other top-down and bottom-up inputs of somatosensory and auditory origin exist in the adult and exhibit similar preference for distinct LCIC compartments (Wiberg and Blomqvist, 1984; Saldaña and Merchán, 1992; Saldaña et al., 1996; Winer et al., 1998; Zhou and Shore, 2006; Bajo et al., 2007; Torii et al., 2013; Stebbings et al., 2014; Lesicko et al., 2016). With such multi-level convergence arising from multiple sensory systems, several mechanisms likely work in concert to instruct LCIC topographic mapping. If pioneer work in unimodal systems is any indication (McLaughlin and O’Leary, 2005; Miller et al., 2006; Luo and Flanagan, 2007; Cang et al., 2008; Triplett et al., 2009, 2011; Imai et al., 2010; Cang and Feldheim, 2013; James et al., 2020), it is probable that precise IC map formation relies upon both activity-dependent and activity-independent mechanisms. Complex mapping along multiple axes normally involves a combination of molecular guidance cues, patterned neural activity, and axon-axon competition. Unlike the neighboring CNIC that exhibits continuous map features (Fathke and Gabriele, 2009), molecular gradients (Gabriele et al., 2011; Wallace et al., 2013, 2016; Cramer and Gabriele, 2014), and cochlear-generated spontaneous events, the LCIC is discretely-organized (Lesicko et al., 2016; Lamb-Echegaray et al., 2019) with matching guidance patterns (Gay et al., 2018; Stinson et al., 2021) and intrinsically generated activity likely arising from multiple sources (Tritsch and Bergles, 2010; Wang and Bergles, 2015; Nakazawa et al., 2020; Babola et al., 2021).

In lieu of gradients, Eph-ephrin guidance molecules are transiently expressed in the LCIC in discontinuous patterns that correlate temporally and spatially with its emerging modularity and developing afferent arrays. Just prior to, and throughout the period that somatosensory and auditory projections separate within the LCIC, EphA4 and ephrin-B2 expression is patchy and overlaps GAD-positive modular domains (Gay et al., 2018). Ephrin-B3 expression, on the other hand, is complementary and localized to the surrounding CR-positive matrix (Stinson et al., 2021). After inputs have fully segregated (P12), expression of these signaling molecules is sharply downregulated. It is likely that discrete Eph-ephrin patterns provide necessary positional information for establishing rough somatosensory vs. auditory terminal compartments. Additional experimentation including loss-of-function studies in mice are needed to tease how specific Eph-ephrin interactions instruct modality-specific LCIC compartments. Once a course map is molecularly established, neural activity often provides subsequent fine-tuning or refinement, such that inputs with correlated activity patterns target similar neuronal subsets, more so than those exhibiting unrelated activity (Butts and Rokhsar, 2001; Debski and Cline, 2002; Cang et al., 2008; Triplett et al., 2012). Applying these insights from other systems/structures to the LCIC model, one might hypothesize that rhythmic spontaneous bursting in the auditory pathway is key for aligning multiple auditory maps within its matrix. Similarly, evidence for early somatotopic activity triggered by spontaneous muscle twitches (Khazipov et al., 2004; Luhmann et al., 2016) may prove equally important for the registry of multiple somatosensory inputs that map to its modular domains. Finally, the juxtaposition of multimodal projection domains (modular/matrix) carrying immature activity presumed to be uncorrelated (i.e., of either somatosensory or auditory origin) may serve to further demarcate LCIC compartmental boundaries. Further investigations that replicate the present tracing experiments and quantify the degree of projection overlap in mice lacking necessary Eph-ephrin cues, structured activity, or both, are needed to determine how these distinct afferent sets achieve proper alignment. Such data will be instrumental in developing future computational models that make LCIC mapping predictions based on various experimental conditions, similar to those already being successfully employed in other analogous systems (Reber et al., 2004; Yates et al., 2004; Tsigankov and Koulakov, 2006; Owens et al., 2015; Savier et al., 2017; Savier et al., 2020).

Besides the potential mechanisms mentioned above, molecules and signaling canonically associated with immune function have been implicated of late as important regulators of developmental pruning and synaptic plasticity (Faust et al., 2021). In particular, microglia as the resident macrophages of the CNS have received significant attention for their presence and significant roles in sculpting newly formed networks. Microglial selective engulfment of extraneous or underutilized contacts has been described in several developing systems and appears to be activity-dependent (Paolicelli et al., 2011; Hoshiko et al., 2012; Schafer et al., 2012; Stephan et al., 2012; Pagani et al., 2015; Hong et al., 2016; Thion and Garel, 2017). Among others, the classical complement cascade (C3–CR3) and fractalkine (CX3CL1–CX3CR1) signaling figure prominently in microglial cell (MGC) recruitment to areas of active circuit assembly and tagging of less active connections for subsequent phagocytic removal. Disruption of MGC function/signaling often results in sustained deficits or delayed maturation of synaptic function, as well as impairments in projection refinement. Compromised complement signaling affects the normal segregation of ipsi- and contralateral retinogeniculate inputs, with significant maintenance of initial projection overlap (Stevens et al., 2007; Schafer et al., 2012). In somatosensory cortex, fractalkine signaling controls entry of MGCs into barrel fields and CX3CR1-deficiency delays functional maturation of its connectivity (Hoshiko et al., 2012). Microglia appear to also perform similar functions in the developing auditory brainstem, evidenced by significant pruning deficits and abnormal auditory brainstem responses when MGCs are pharmacologically depleted (Milinkeviciute et al., 2019, 2021a). While pruning at the calyx of Held is unaffected in fractalkine receptor mutants, these mice express increased glycinergic synaptic markers in the auditory brainstem, suggesting an important role for CX3CL1–CX3CR1 crosstalk in sculpting immature inhibitory connections (Milinkeviciute et al., 2021b). Recent work from our lab shows microglia indeed invade the nascent IC and that compromised fractalkine signaling delays MGC occupancy of its emerging modules (Brett et al., 2022). The present study quantifying the normal development and segregation of multimodal LCIC inputs serves as important baseline for ongoing experimentation aimed at determining whether MGCs and their established signaling pathways influence selective pruning at the level of the midbrain, and if compromised, whether multisensory distributions fail to segregate. If true, such lack of refinement and sustained overlap might overwhelm LCIC circuits. Instead of first segregating sensory information so that it can then be shared locally (Lesicko et al., 2020) before being passed on for further integration at the level of the deep superior colliculus (SC, among other multisensory centers), unrefined network configurations may preclude the LCIC from performing its function as a staging area for meaningful merging of the senses.

In conclusion, our experiments demonstrate that modality-specific LCIC projection patterns segregate over an early postnatal critical period. Beyond sharing a similar window for circuit assembly and mapping adjustments defined for other uni- and multimodal structures (Hoshiko et al., 2012; Schafer et al., 2012; Triplett et al., 2012), it appears the spatial registry of converging LCIC maps may too be governed by a combination of chemical labeling, neural activity, and competitive influences. Understanding the mechanisms that instruct its early plasticity and functional maturation is vital for gaining insights into certain neurodevelopmental conditions, such as autism spectrum disorders and schizophrenia. Both are characterized by gross errors in pruning (under- and over-pruning, respectively; Lehrman et al., 2018; Faust et al., 2021) and multimodal processing deficits that likely contribute to a host of behavioral consequences (Kwakye et al., 2011; Stevenson et al., 2014a, b; Brandwein et al., 2015; Tseng et al., 2015; Robertson and Baron-Cohen, 2017). Through further study of multisensory map alignments and the dynamic processes that guide their selective refinement, we may better understand the factors underlying these debilitating disorders and perhaps uncover new means for their successful treatment.

## Data Availability Statement

The raw data supporting the conclusions of this article will be made available by the authors, without undue reservation.

## Ethics Statement

The animal study was reviewed and approved by James Madison University’s Animal Care and Use Committee (approval number, 20-1421).

## Author Contributions

JW, EK, JC, and MG all contributed to the presented experiments. JW, EK, and JC performed all tissue processing, associated imaging, and data sampling. MG performed data quantification, prepared all figures, and wrote the manuscript. All authors contributed to the article and approved the submitted version.

## Conflict of Interest Statement

The authors declare that the research was conducted in the absence of any commercial or financial relationships that could be construed as a potential conflict of interest.

## Publisher’s Note

All claims expressed in this article are solely those of the authors and do not necessarily represent those of their affiliated organizations, or those of the publisher, the editors and the reviewers. Any product that may be evaluated in this article, or claim that may be made by its manufacturer, is not guaranteed or endorsed by the publisher.

## Footnotes

^1^ www.clarechemical.com

^2^ https://imagej.net/

## Supplementary Material

Supplementary Figure 1 Two additional P12 cases **(A–D,E–H)** showing complementary, modality-specific LCIC afferent patterns. Somatosensory terminals (red) are concentrated within GAD-positive modules (green, dashed contours), while auditory inputs (cyan) distribute throughout the surrounding matrix. Projections have essentially segregated at this age (i.e. minimal overlap) with patterns in keeping with those described in the adult, suggesting the end of an early critical period for shaping discrete, multisensory LCIC input arrays. Scale bars = 100 μm.
