# Supplementary figures and images for: Segregation of Multimodal Inputs Into Discrete Midbrain Compartments During an Early Critical Period
Source: Front Neural Circuits. 2022 Apr 7;16:882485. doi: 10.3389/fncir.2022.882485 (PMC9021614; doi:10.3389/fncir.2022.882485)

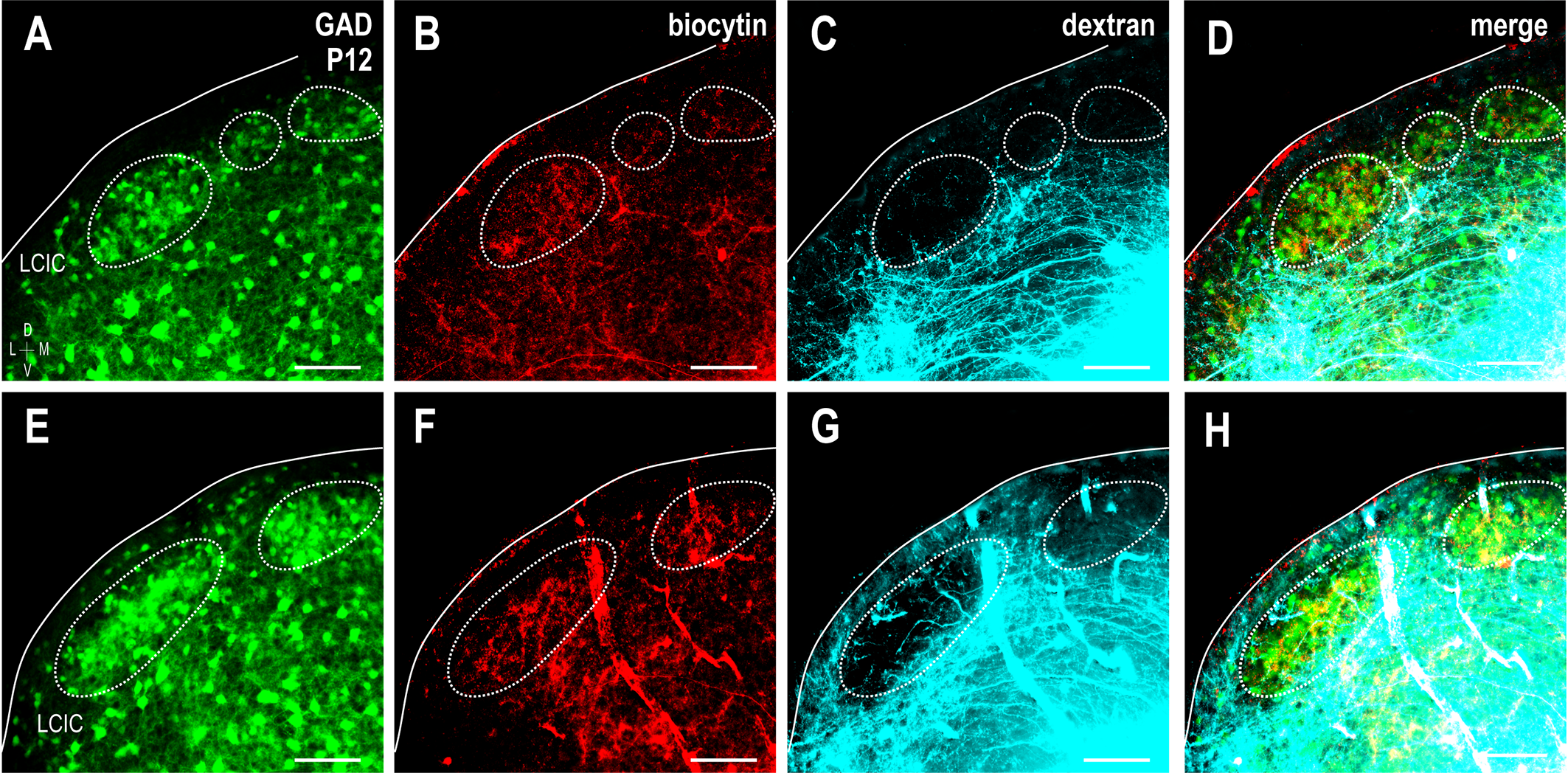

Supplement: Supplementary Figure 1 — Two additional P12 cases (A–D,E–H) showing complementary, modality-specific LCIC afferent patterns. Somatosensory terminals (red) are concentrated within GAD-positive modules (green, dashed contours), while auditory inputs (cyan) distribute throughout the surrounding matrix. Projections have essentially segregated at this age (i.e. minimal overlap) with patterns in keeping with those described in the adult, suggesting the end of an early critical period for shaping discrete, multisensory LCIC input arrays. Scale bars = 100 μm. [file Image_1.TIF]
